# Reason for euthanasia in dogs with urothelial carcinoma treated with chemotherapy or radiation therapy or both: A retrospective observational study

**DOI:** 10.1111/jvim.16994

**Published:** 2024-02-05

**Authors:** Charly McKenna, Valerie J. Poirier, Michelle L. Oblak, Stephanie Nykamp, Anthony J. Mutsaers

**Affiliations:** ^1^ Biomedical Sciences, Ontario Veterinary College University of Guelph Guelph Ontario Canada; ^2^ Clinical Studies, Ontario Veterinary College University of Guelph Guelph Ontario Canada

**Keywords:** bladder cancer, canine, transitional cell carcinoma, urinary obstruction

## Abstract

**Background:**

Clients want to know the ultimate cause of death in their pet after cancer treatment. The cause of euthanasia and investigation of urinary obstruction in treated dogs with urothelial carcinoma (UC) has not been specifically reported in veterinary literature.

**Hypothesis/Objectives:**

Our hypothesis was that the majority of treated dogs with UC are euthanized secondary to primary tumor factors, such as urinary obstruction.

**Animals:**

Fifty‐nine client‐owned dogs diagnosed with UC.

**Methods:**

Retrospective observational study on clinical signs and disease at euthanasia of dogs with UC treated by radiation therapy or chemotherapy or both.

**Results:**

The median overall survival time (OST) of all dogs was 339 days (range, 17‐1996; 95% confidence interval [CI], 185‐392; interquartile range [IQR], 112‐505). Of dogs deemed to have been euthanized because of UC (50/59, 85%), the primary cause was considered to be local progression in 31/50 (62%), most often because of perceived complete or partial urinary obstruction (24/31, 77%). No variables were found to be predictive of urinary obstruction. The overall documented metastatic rate was 56%. In dogs euthanized because of UC, metastasis was deemed to be the cause in 19/50 (38%) dogs.

**Conclusions and Clinical Importance:**

Regardless of the type of treatment, UC in dogs has a poor prognosis and there is a continuing need to improve treatments that focus on local control of the primary tumor, given its high contribution to the decision for euthanasia. Proactive management to avoid the high frequency of urinary obstruction may be worthy of future investigation.

**Abbreviations**
FNA: fine needle aspirate
MILN: medial iliac lymph node
NSAID: nonsteroidal anti‐inflammatory drug
OST: overall survival time
OVC‐HSC: Ontario Veterinary College Health Sciences Centre
RT: radiation therapy
UC: urothelial carcinoma

## 1 INTRODUCTION

Carcinomas involving the bladder and urethra account for approximately 2% of reported cancers in dogs.^1^, ^2^ Dogs with urothelial carcinoma (UC) typically have nonspecific lower urinary tract clinical signs, including stranguria, hematuria, or pollakiuria.^2^, ^3^, ^4^, ^5^ Dogs with advanced cases of UC may be presented for signs consistent with local progression (eg, bladder rupture, distended and painful abdomen, urinary obstruction) or metastasis (eg, enlarged lymph nodes, lameness, respiratory difficulty, weight loss) or both.^4^, ^5^, ^6^

Most frequently, UC is diagnosed in both the bladder and urethra (>50% of cases).^1^, ^2^ Tumors may also develop or progress locally in the bladder, urethra, prostate, ureters, and kidneys.^1^, ^2^, ^5^, ^6^, ^7^, ^8^ Local tumor progression can decrease urine flow, leading to urinary obstruction, and ultimately, hydronephrosis.

No definitive standard of care has been established for the treatment of UC in dogs. Local treatment can consist of surgery,^9^, ^10^, ^11^ radiation therapy (RT),^3^, ^12^, ^13^, ^14^, ^15^, ^16^, ^17^, ^18^ laser treatment,^19^, ^20^ prostatic embolization^7^ or chemoembolization,^8^ urinary catheterization, or urethral stenting when pathologic urinary obstruction occurs.^2^, ^4^, ^20^ Systemic treatment usually consists of a nonsteroidal anti‐inflammatory drug (NSAID)^21^, ^22^ often combined with chemotherapy (eg, mitoxantrone, carboplatin, doxorubicin, vinblastine, chlorambucil).^5^, ^23^, ^24^, ^25^, ^26^, ^27^, ^28^, ^29^ Overall, UC is considered to have a poor response to chemotherapy with an objective response rate to most chemotherapeutic regimens <40%.^5^, ^6^, ^24^, ^29^ Combination or rescue chemotherapy protocols may prolong survival time in dogs with UC.^28^, ^30^ Dogs that received ≥3 chemotherapy drugs experienced longer median survival time than dogs that received 1 or 2 drugs (402 days compared to 190 days, respectively).^30^ When lymph node metastasis had occurred, these dogs experienced significantly shorter survival times (47 days).^30^ Additionally, use of metronomic PO chlorambucil after prior treatment failure resulted in a median survival time of 221 days, which was negatively impacted by distant metastases, as well as prior cyclooxygenase inhibitor treatment and urethral involvement.^28^

Although some variation in survival times occurs based on the treatment protocol used, regardless of treatment, dogs with UC generally are euthanized because of their disease (either local or metastatic) within 1 year.^1^, ^2^, ^3^, ^4^, ^5^, ^6^ It is generally considered that without treatment, most dogs die or are euthanized because of complications associated with local disease progression.^2^, ^4^ However, when the primary tumor is treated, metastatic disease occurs frequently in >50% of reported cases.^1^, ^2^, ^3^, ^4^, ^31^ As evidenced by the number of treatment options described above, local disease control methods are becoming increasingly sophisticated, and it may be challenging to predict the likelihood of subsequent life‐limiting metastatic disease for owners, because the cause of death has not been a primary focus of prior reports of UC in dogs. In a clinical trial of mitoxantrone and piroxicam, 6 of 55 dogs had metastatic disease at the time of presentation, whereas 14 of 49 dogs with complete data had metastatic disease at the time of death.^6^ Deaths in 28 of the 49 dogs were related to UC.^6^ In a study of radiation therapy, mitoxantrone and NSAID, of 20 dogs with locoregional failure and documentation of cause of euthanasia, 15 died of locoregional progression, 4 of metastatic disease, and 1 of both.^18^ In 12 dogs that had locoregional control at the time of death, 5 dogs died of metastatic disease and in 7 dogs death was deemed not to be tumor‐related.^18^ Given the sporadic documentation of cause of death in prior studies, coupled with the possibility that the distribution of metastatic versus local failure may be changing with more advanced treatments, our purpose was to retrospectively investigate the cause for euthanasia in dogs with UC treated at a single institution. Our hypothesis was that failure to control local disease (primarily urinary obstruction) would be the leading cause of euthanasia.

## 2 MATERIALS AND METHODS

### 2.1 Case selection

**FIGURE 1:**
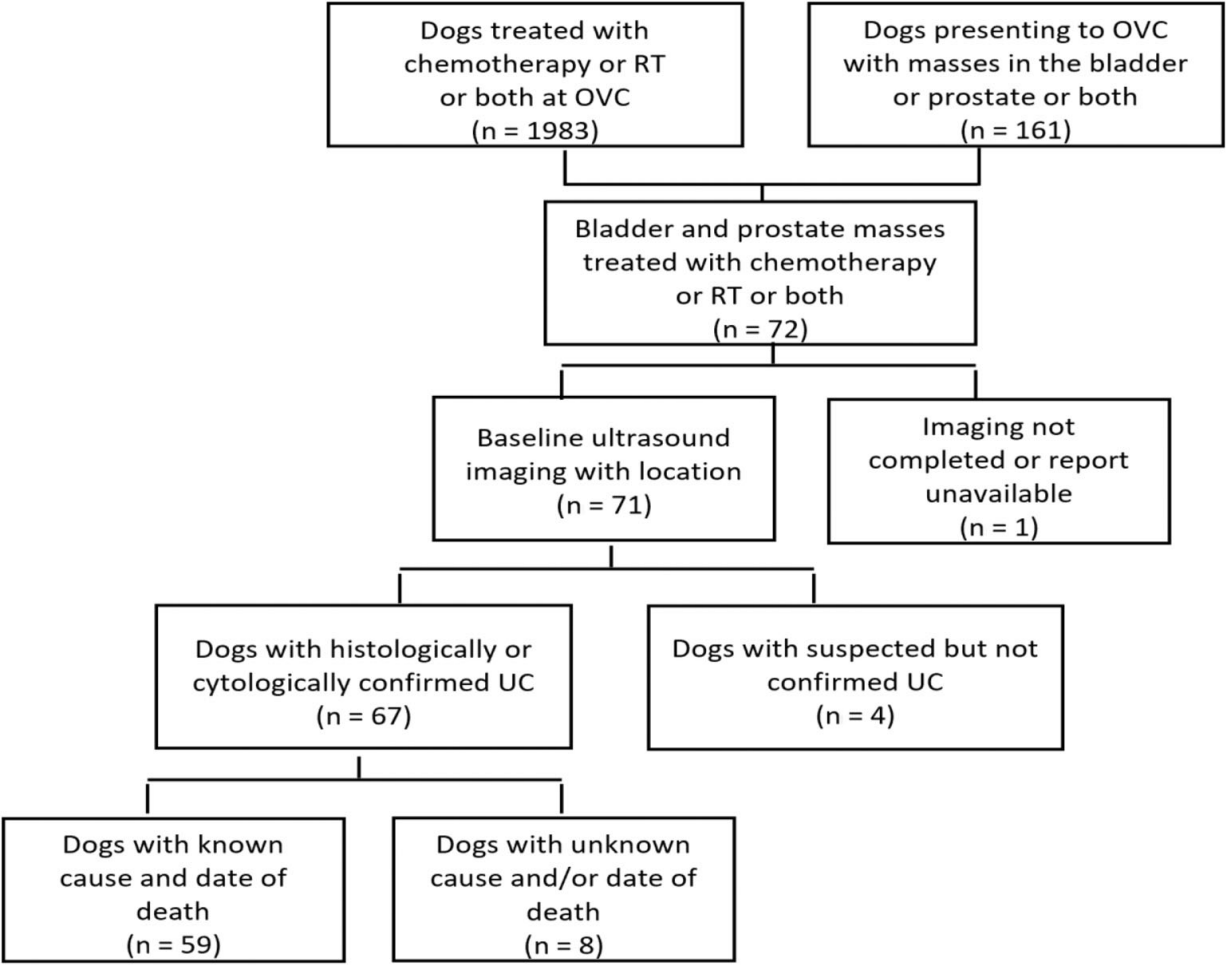
Summary of included and excluded dogs with cancer that received treatment (chemotherapy or radiation therapy [RT]) or both for confirmed urothelial carcinoma (UC). A total of 59 dogs met the inclusion criteria for this study.

Medical records from the Ontario Veterinary College (OVC) Health Sciences Centre Companion Animal Hospital from January 1, 2011 to March 3, 2020 were evaluated (Figure 1). Inclusion criteria were dogs with cytologically or histologically confirmed UC, treated with chemotherapy or RT or both that had baseline abdominal ultrasound examination performed by a board‐certified radiologist and where the date and cause of death were known. Dogs treated only with NSAIDs were excluded.

### 2.2 Medical records review

Data collected from the medical record included patient signalment (breed, sex, age), clinical signs and method of diagnosis, blood test results including CBC and serum biochemistry, original and follow‐up radiographic and ultrasound imaging findings (tumor location, presence of metastatic disease), treatment types (surgery, chemotherapy [drug, number of treatments] or RT [fractionation and dose] or both and NSAID [yes/no and type]), reported emergency visits, cause of death and presenting complaint at time of death. In cases for which additional follow‐up was necessary, referring veterinarians were contacted by telephone or email or both.

### 2.3 Classification of disease location at diagnosis

The location of the disease at diagnosis was based on the initial ultrasound report and was classified into 4 categories: bladder only, prostate only, urethra only and multifocal. Tumors in the bladder were further subdivided into 4 categories: apex, ventral neck, trigone, and diffuse. The location was determined based on review of the ultrasound report and images by a single board‐certified radiologist (SN).

### 2.4 Classification of death and urinary obstruction

The cause of death was classified into 3 categories: UC local disease (urinary obstruction or local disease progression), UC metastatic disease and non‐UC related (concurrent diseases considered unrelated to UC). Categorizing the cause of death of dogs included in the study involved a systematic approach that considered various factors and clinical information. This approach included reviewing the medical records, diagnostic test results and imaging reports, and any communication notes between clients and veterinary staff preceding the euthanasia appointment. Records from both the specialty hospital and referring veterinarian were evaluated when dogs were not euthanized at OVC. If cause of death was not obvious from the available medical records, follow‐up by phone or email with the primary veterinarian or specialty clinician was completed. Urinary obstruction was defined anatomically as urethral, ureteral or both based on imaging, clinical signs, laboratory reports, necropsy finding (when available) or some combination of these. Azotemia documented on serum biochemistry was not deemed to be caused by urinary tract obstruction without corroborating evidence from imaging studies. Local disease was considered the cause of euthanasia when obstructive signs were documented. Metastasis was considered the cause of euthanasia when it was clinically documented and there was no clinical evidence of primary tumor growth or obstruction leading to impaired quality of life, as ascertained by clinical record review.

### 2.5 Statistical analysis

Overall survival time (OST) was defined as the first day of treatment until death of any cause. The Kaplan‐Meier method including Hall‐Wellner confidence bands homogeneity tests were used to calculate median OST, 95% CI and to test for homogeneity. In addition, variables assessed for potential contribution to urinary obstruction (sex, treatment, overall location, and location within the bladder) were evaluated by odds ratio testing. A *P*‐value <.05 was considered significant. Commercially available statistical software (SAS, release 3.8, SAS Institute, Inc, Cary, NC) was used for analysis.

## 3 RESULTS

Fifty‐nine dogs met inclusion criteria for the study (Figure 1). Dogs and clinical presentation characteristics were typical for UC. Median age was 10 years (range, 4‐14 years). Dogs consisted of 27 spayed females (46%), 1 intact female (2%), 30 neutered males (51%), and 1 intact male (2%). There were 12 mixed breed dogs (20%). The most common purebred dogs in the study were West Highland white terrier (n = 8, 14%), Shetland sheepdog (n = 5, 8%), Beagle (n = 3, 5%), and Yorkshire terrier (n = 3, 5%). Presenting complaints included hematuria (n = 33, 56%), stranguria (n = 25, 42%), urinary incontinence (n = 9, 15%), obstruction causing inability to urinate (n = 4, 7%), polyuria and polydipsia, (n = 11, 19%), inappetence (n = 2, 3%) and weight loss (n = 1, 2%).

Diagnosis of UC was confirmed histologically in 46 dogs (78%), by catheter‐obtained biopsy sample in 31 dogs (68%), surgical tumor reduction or removal and biopsy at the time of cystotomy for bladder calculi removal in 3 dogs each (7%). In the remaining 13 dogs (22%), UC was confirmed cytologically by urine sediment examination in 9 dogs (69%), fine needle aspirate (FNA) of the prostate gland in 2 dogs (15%), FNA of the lymph nodes and prostatic wash cytology in 1 dog each (8%). None of the included dogs were diagnosed using a commercially available urinary antigen test or CADET BRAF test (Antech Diagnostics, USA).

All 59 dogs had thoracic radiography and abdominal ultrasound examination performed at the time of diagnosis. Nine dogs (15%) had evidence of metastasis (medial iliac lymph node [MILN], n = 3; lungs, n = 4; MILN and sacral lymph node, n = 1; and cutaneous nodules, liver, and lungs, n = 1). The primary tumor location was bladder only (38/59, 64%), urethra only (11/59, 19%), prostate only (5/59, 8%) and multifocal (5/59, 8%). Tumor locations within the bladder were trigone (15/38, 39%), diffuse (9/38, 24%), apex (9/38, 24%), and ventral neck (5/38, 13%).

**FIGURE 2:**
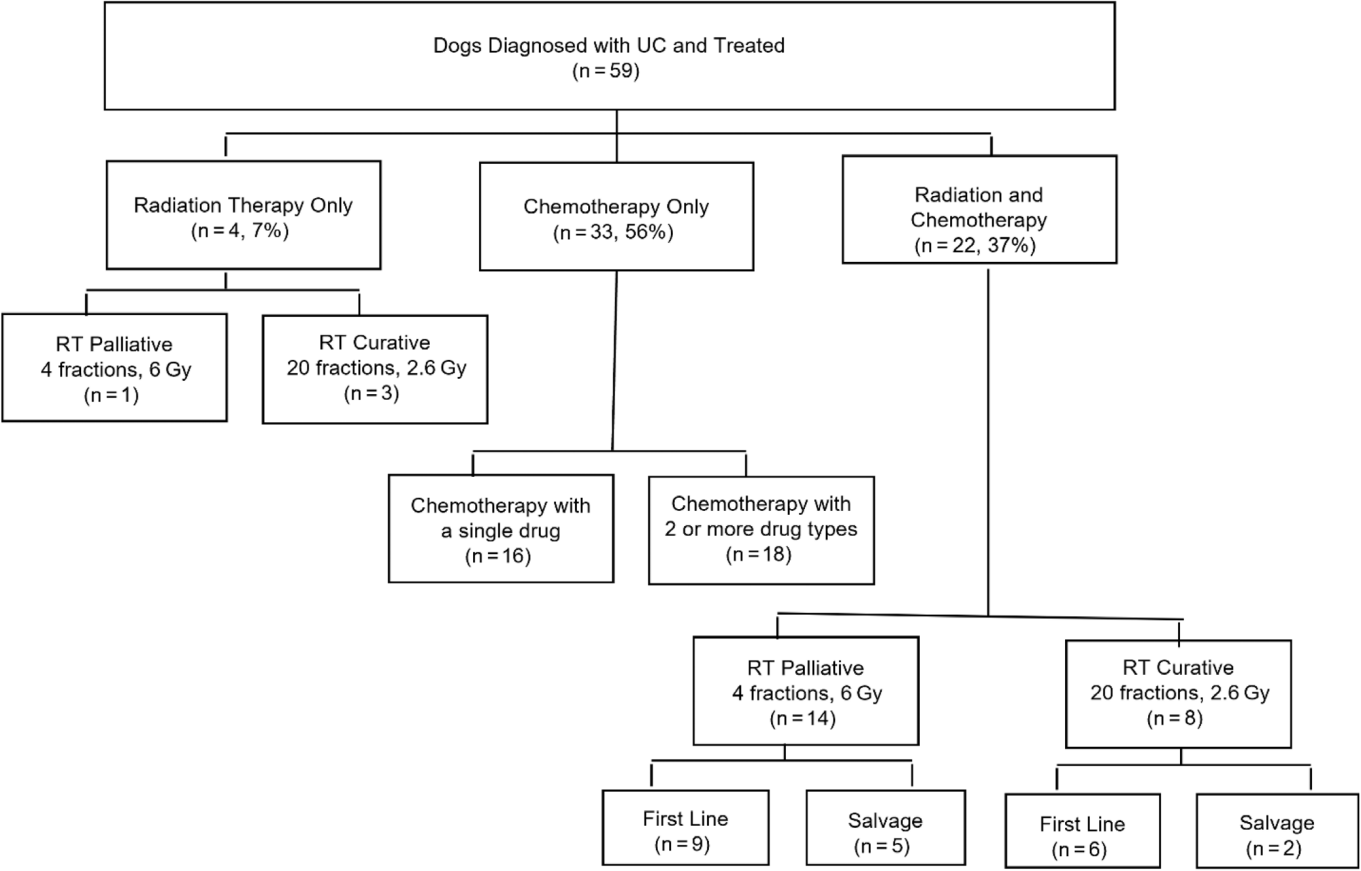
Summary of treatment types (chemotherapy and radiation therapy [RT]) for 59 dogs diagnosed with urothelial carcinoma (UC). Chemotherapy consisted of single drug protocols only, and thus multiple drug types indicated use of new single drug protocols in the second line setting and beyond. The use of NSAID was common in many protocols and not specifically included in the figure.

All 59 dogs were treated for UC (Figure 2); 55 dogs (93%) received chemotherapy, of which 22 dogs (37%) also received RT and 4 dogs (7%) also underwent surgery (partial cystectomy [n = 3] or partial prostatectomy [n = 1]). Four dogs (2%) received only RT. Chemotherapy protocols included 6 different drug types administered as single‐agent protocols: mitoxantrone, vinblastine, carboplatin and doxorubicin administered IV and chlorambucil and toceranib phosphate (Palladia, Zoetis, USA) administered PO. The median number of IV chemotherapy doses was 5 (range, 1‐18). Furthermore, 47 dogs (80%) were treated with an NSAID (meloxicam [n = 42] or piroxicam [n = 5]).

Forty‐seven dogs (80%) had at least 1 unscheduled emergency visit, either to the OVC, referring veterinarian, or other specialty hospitals. These visits did not result in euthanasia and were most often for complaints regarding partial or complete urinary obstruction (25/47, 53%). Emergency visits occurred either during the radiation or chemotherapy treatment protocol (nonurinary obstruction, n = 14; urinary obstruction, n = 5) or after completion of treatment (nonurinary obstruction, n = 8; urinary obstruction, n = 20).

After baseline staging (based on abdominal ultrasound examination and thoracic radiography), all 59 dogs had subsequent abdominal ultrasound examinations (median, 3; range, 1‐20) and 20 dogs had thoracic radiography (median, 2; range, 1‐5). The intervals of follow‐up abdominal ultrasounds from initial treatment included ≤3 months (n = 54), 3‐6 months (n = 31), 6‐9 months (n = 17), 9‐12 months (n = 16), 12‐18 months (n = 15), 18‐24 months (n = 6) and >2 years (n = 5). Before euthanasia, 10 dogs had abdominal ultrasound examinations within 2 weeks and 23 dogs within 1 month. Necropsy was performed on 7 of the 59 dogs (12%). Although numerous additional findings were documented on necropsy, none of the conclusions regarding cause of euthanasia on the 7 necropsied dogs contradicted the conclusions drawn from medical record data obtained antemortem. Two dogs were deemed to have been euthanized because of metastatic disease (from systemic vascular thrombosis and tumor emboli causing acute congestive heart failure and lung metastases in a dog with stable primary tumor), 4 because of local disease, and 1 for nontumor related reasons (intervertebral disc disease).

At the time of diagnosis, 4 dogs (4/59, 8%) had hydronephrosis or hydroureter or both. During follow‐up abdominal ultrasound examination, hydronephrosis or hydroureter or both were documented in an additional 13 dogs, leading to a total of 17 dogs (29%) that developed this condition. Most occurrences of hydronephrosis or hydroureter or both were associated with progression of UC, but in 3 dogs hydroureter was attributed to RT adverse effects and resolved with time. Hydronephrosis or hydroureter‐induced renal failure was the cause of euthanasia in 3 dogs (5%).

At diagnosis, 4 dogs (4/59, 8%) had partial or complete urinary tract obstruction. During follow‐up, complete or partial urinary obstruction was documented in an additional 25 dogs, for a total of 29 dogs (49%) that developed obstruction at some point. During or after treatment, 3 dogs (4%) had urethral stents placed because of complete obstruction. Local disease progression was the cause of euthanasia in 31 dogs (62%; Figure 4). Most of these dogs (41%, 24/31) were euthanized because of urinary tract obstruction diagnosed based on imaging or clinical signs including abdominal pain and inability to urinate (Table 1). Necropsy in 2 of the obstructed dogs confirmed clinically‐diagnosed obstruction and marked invasion into neighboring structures (urethra, ureters, trigone, and prostate) was noted.

**TABLE 1:** Presenting complaints based on client assessment at end‐of‐life appointment for 50 dogs euthanized because of urothelial carcinoma (UC).

Presenting complaint	Dogs euthanized because of UC (n = 50)	Dogs euthanized because of local disease (n = 31)	Dogs euthanized because of metastatic disease (n = 19)
Abdominal pain	n = 19, 38%	n = 16, 52%	n = 3, 16%
Inability to urinate (obstruction)	n = 19, 38%	n = 18, 58%	n = 1, 5%
Inappetence	n = 21, 42%	n = 13, 42%	n = 8, 42%
Lethargy	n = 17, 34%	n = 10, 32%	n = 7, 37%
Stranguria	n = 12, 24%	n = 12, 39%	–
Hematuria	n = 12, 24%	n = 10, 32%	n = 2, 11%
Progressive disease (diagnosed on imaging)	n = 15, 48%	n = 10, 32%	n = 5, 26%
Weight loss	n = 12, 24%	n = 7, 23%	n = 5, 26%
Lameness	n = 5, 10%	–	n = 5, 26%
Decreased or poor quality of life	n = 11, 22%	n = 7, 23%	n = 4, 21%
Nonambulatory	n = 5, 10%	n = 1, 3%	n = 4, 21%
Incontinence	n = 9, 18%	n = 7, 23%	n = 2, 11%
Lethargy	n = 4, 8%	n = 1, 3%	n = 3, 16%
Respiratory difficulty	n = 5, 10%	n = 2, 6%	n = 3, 16%
Distended bladder	n = 4, 8%	n = 4, 13%	–
Nausea	n = 4, 8%	n = 2, 6%	n = 2, 11%
Polyuria and polydipsia	n = 6, 12%	n = 4, 13%	n = 2, 11%
Not sleeping	n = 1, 2%	–	n = 1, 5%
Coughing	n = 1, 2%	–	n = 1, 5%
Intermittent fever	n = 1, 2%	–	n = 1, 5%
Anxiety	n = 3, 6%	n = 3, 10%	–
Renal failure	n = 3, 6%	n = 3, 10%	–

At diagnosis, 9 dogs (9/59, 15%) had evidence of metastasis. During follow‐up imaging or necropsy (n = 2), metastasis was confirmed in an additional 24 dogs, leading to a total of 33 dogs (56%). An additional 3 dogs (5%) had suspected metastases based on clinical signs (inappetence, lethargy, respiratory difficulty), but these findings were not confirmed with diagnostic imaging or necropsy. There were 21 dogs (64%) with single sites of metastasis and 12 dogs (36%) with multiple sites. The most common sites of metastasis at euthanasia were lymph nodes (n = 23, 22%), lung (n = 12, 36%), bone (most often humerus; n = 6, 18%), liver (n = 2, 6%), and abdominal cavity, cutaneous masses, eye, heart, and adrenal gland (n = 1 each, 3%). One dog developed abdominal seeding of the tumor after FNA. This dog also had a small nodule in the SC tissue along the caudoventral abdomen, at the caudal aspect of a previous cystotomy incision. The overall documented metastasis rate in our population was 61%. Metastasis was the cause of euthanasia in 19 dogs (32%). Necropsy in 2 of these dogs confirmed clinically‐diagnosed (pulmonary edema, lethargy, painful abdomen, respiratory difficulties) metastases to the lungs.

**FIGURE 3:**
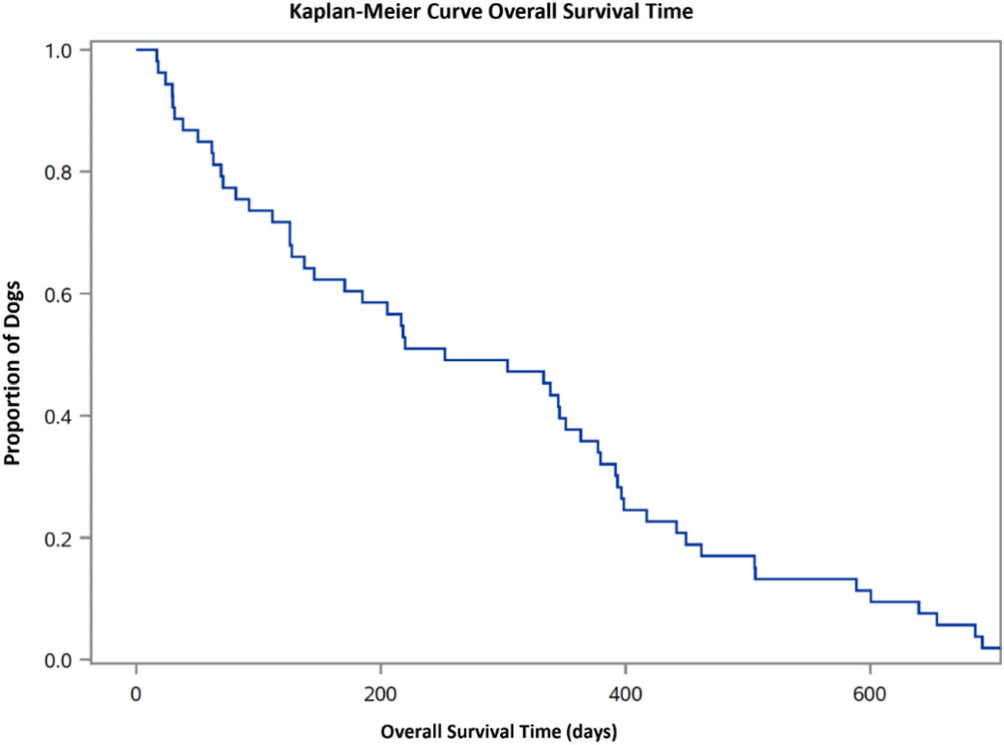
Kaplan‐Meier overall survival time (OST) of 59 treated dogs with urothelial carcinoma (UC). All dogs were deceased at the conclusion of data collection, median OST was 339 days (95% CI, 185‐392; IQR, 112‐505; range, 17‐1996).

All dogs had died or were euthanized at the conclusion of data collection for the study. Median OST was 339 days (range, 17‐1996; 95% CI, 185‐392; IQR, 112‐505; Figure 3). Because the setting and treatments differed (confirmed by Hall‐Wellner confidence bands and homogeneity tests, *P* = .61), no further survival analyses were performed.

Nine dogs (15%) died or were euthanized because of causes unrelated to UC based on review of medical records (5/9) or follow‐up with referral veterinarians (4/9). Necropsy was performed in 2 of these dogs and documented the extent of disease. For the remaining 7 dogs, without necropsy it was not possible to determine the extent of disease and therefore it was possible the tumor still could have contributed to the decision to euthanize these dogs. Categorization was based on judgment of the clinician in these cases based on medical record information and veterinary communications. Causes of euthanasia in these cases were decreased mobility (n = 2; 1 dog had a necropsy‐confirmed severe degenerative intervertebral disk disease), concurrent neoplasms (hepatocellular carcinoma) and masses within spleen and liver (n = 2), chronic kidney disease before development of UC (n = 1), respiratory arrest of unknown cause (n = 1), cognitive changes (n = 1), sepsis secondary to a clinical trial drug (n = 1; septicemia confirmed at necropsy) and suspected gastric ulceration (n = 1).

**FIGURE 4:**
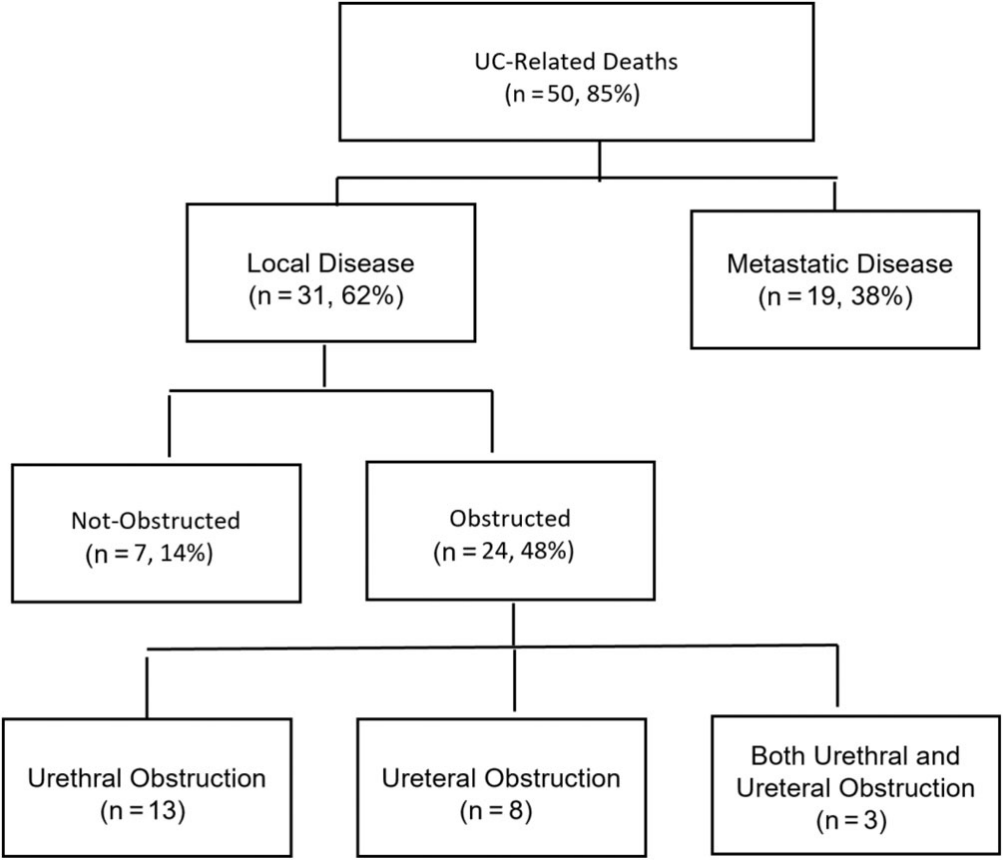
Comparison of dogs (n = 50) euthanized because of urothelial carcinoma (UC).

The remaining 50 euthanized dogs (85%) were deemed to have been euthanized because of UC based on medical records (22/50), or follow‐up with referral veterinarians (28/50; Figure 4). Of the 24 dogs with partial or complete urinary obstruction, 13 dogs (54%) had confirmed metastases and 1 dog (4%) had suspected metastases. Azotemia was observed in 42% of the obstructed dogs at time of obstruction. In the 3 dogs that had stents placed after urethral obstruction, the cause for euthanasia was not local but attributed to lung metastasis, bone metastasis, and suspect gastric ulceration at 384, 37 and 268 days, respectively, after stent placement. All variables analyzed for association with urinary obstruction (treatment: RT vs non‐RT, sex, overall location, and bladder location) were not significant.

Specific presenting complaints leading to euthanasia were determined for all cases included in the study. The most frequent reasons for euthanasia were inappetence (n = 21), inability to urinate because of obstruction (n = 19), abdominal pain (n = 19), and lethargy (n = 17; Table 1).

## 4 DISCUSSION

We wished to investigate the degree to which local disease contributes to cause of death in dogs with UC, even in those with longer survival times. The median survival time in our study population was 339 days, and most of the dogs were euthanized because of local progression (31/50, 62%). Most of these dogs were deemed to have complete or partial urinary obstruction (24/31, 77%) at the time of euthanasia. In addition, >80% of dogs were presented to an emergency hospital for at least 1 visit, not related to euthanasia, and 53% of these visits were for partial or complete urinary obstruction. No variables (treatment, sex, tumor location) were strongly associated as predictive of urinary obstruction in the included population.

The 59 dogs included in our study had signalment (breed, sex, age) and primary complaints (hematuria, stranguria) consistent with earlier studies of UC.^2^, ^3^, ^13^, ^32^ Confirmation of UC diagnosis was completed primarily (78%) by means of histology. Fine needle aspirate cytology of UC tumors or metastatic sites was performed in only 5% of cases, differing from previous studies where FNA was the main diagnostic method (91% of 115 dogs).^3^ This difference is likely because of increased recognition of the propensity of these tumors to seed needle tracts after such manipulations.^33^, ^34^ When added to the 9 cases that had a diagnosis based on urine sediment cytology, 12 cases were included based on cytologic diagnosis. In our cohort of dogs, abdominal seeding after FNA was confirmed in 1 case. This seeding presented as a small nodule within the SC tissue along the caudoventral abdomen, at the caudal aspect of a previous cystotomy incision. The reason for this dog's euthanasia was suspected metastases to the abdomen (progressive) and lungs (new), but metastasis was not confirmed on diagnostic imaging.

The moderate rate of metastasis (61%) in our study is in agreement with previous reports.^1^, ^2^, ^18^, ^31^ Because only a small number of dogs (7/59) had necropsy performed, the metastatic rate may have been underestimated. In addition, although the number of dogs in our study with lung metastasis (36%) was similar to the previously reported range (17%‐48%),^1^, ^2^, ^3^ the frequency of lung metastasis may be higher because an abdominal ultrasound examination sometimes was prioritized over thoracic radiography for staging and lung lesions may have gone undetected. Emphasizing the importance of regular thoracic radiography, lung metastases were found at necropsy in 3 dogs not previously reported to have metastasis on imaging. The number of dogs with bone metastasis (18%) was slightly higher than the 0%‐14% previously reported in both retrospective and prospective studies.^1^, ^3^, ^35^

Despite the moderate rate of metastasis, local disease progression was most frequently (62%) the cause for euthanasia. In dogs euthanized because of progressive local disease, 24/31 were euthanized because of partial or complete urinary obstruction, with urethral obstruction being most common (16/24, 66%). Approximately half of the cohort of dogs euthanized because of urinary obstruction had imaging or clinical evidence of renal damage (including dilatation of the renal pelvis) or renal failure, possibly caused by intermittent partial or complete obstruction, but some renal changes may have been incidental findings in older dogs. These renal changes emphasize the challenge in determining the cause of death in dogs that may have concurrent disease as opposed to direct consequences of tumor growth. In addition, the presence of metastasis does not necessarily equate to it having been the cause of death. Indeed, we found that although evidence of metastases was observed in 90% of the obstructed dogs, ultimately euthanasia was attributed to local disease in these cases, based on clinical judgment. Interestingly, the opposite also was true in 1 of the dogs classified to have been euthanized based on the documented presence of metastatic disease and no clinical evidence for primary tumor obstruction. This dog actually was found to be have bilateral ureteral obstruction and had been obstructed for some time based on necropsy findings of renal damage. These examples emphasize the complexities of proper attribution of cause of death using clinical data in a retrospective manner.

Our study had several limitations related to its retrospective nature, including the small number of cases, incomplete medical records and lack of standardized treatment and follow‐up. Another limitation is the number of clinicians and therefore variability involved in patient assessment, treatment decisions, restaging intervals, and care. This variability affected case management recommendations, and it is also likely that variability in the interpretation of ultrasound examinations and radiography occurred because not all assessments were performed by the same individual. Furthermore, a large limitation of a retrospective evaluation of perceived cause of death is that the final decision for categorization (local disease, metastatic disease, nontumor‐related) was based on clinical judgment after review of medical records, including client and veterinary communications. Importantly, necropsy was not a required criterion for inclusion in the study, and only 7 cases underwent necropsy, which limits knowledge of the complete distribution of UC and concurrent diseases at the time of death. In addition, the type of imaging modality used to characterize disease burden can influence sensitivity and detectability, and this factor was not controlled. For example, abdominal ultrasonography may inherently underestimate the extent of urethral disease compared to direct visualization with a modality such as urethroscopy or cystoscopy. It is possible that the subjectivity associated with assigning a cause of death based on clinical data led to a bias in categorization, which may be minimized in a prospectively designed study that includes necropsy evaluation.

Our findings illustrate the need to continually improve treatment for dogs with UC and focus more on strategies to improve local control. Future prospective studies that clinically evaluate innovative approaches for controlling local disease should plan careful assessments to document obstruction and determine whether euthanasia results from failure of local control or actual metastatic burden.

## CONFLICT OF INTEREST DECLARATION

Anthony J. Mutsaers serves as Associate Editor for the Journal of Veterinary Internal Medicine. He was not involved in review of this manuscript. No other authors declare a conflict of interest.

## OFF‐LABEL ANTIMICROBIAL DECLARATION

Authors declare no off‐label use of antimicrobials.

## INSTITUTIONAL ANIMAL CARE AND USE COMMITTEE (IACUC) OR OTHER APPROVAL DECLARATION

Authors declare no IACUC or other approval was needed.

## HUMAN ETHICS APPROVAL DECLARATION

Authors declare human ethics approval was not needed for this study.
